# Molecular Epidemiology of Methicillin-Resistant and Methicillin-Susceptible *Staphylococcus aureus* Isolated from the Eye

**DOI:** 10.3109/02713683.2010.534229

**Published:** 2010-12-15

**Authors:** Christine K Hesje, Christine M Sanfilippo, Wolfgang Haas, Timothy W Morris

**Affiliations:** Bausch & Lomb, Inc., Rochester, New York

**Keywords:** Bacterial infection, Community-acquired MRSA, Multi-drug resistance, *spa* typing, Staphylococcal virulence factors

## Abstract

**Purpose:**

Methicillin-resistant *Staphylococcus aureus* (MRSA) strains are commonly classified as hospital-acquired (HA) or community-acquired (CA). Typical HA-MRSA isolates are characterized by multidrug resistance and the SCC*mec* type II cassette, while CA-MRSA isolates are generally susceptible to more drug classes, are often of SCC*mec* type IV, and frequently carry the Panton-Valentine leukocidin (PVL) genes. This study determined the presence of traits characteristic for CA and HA strains in ocular MRSA isolates.

**Materials and Methods:**

Fifty-six recent ocular isolates, consisting of 40 MRSA and 16 methicillin-susceptible *Staphylococcus aureus* (MSSA) comparator strains, were characterized. Minimum inhibitory concentration (MIC) testing was done according to current Clinical and Laboratory Standards Institute guidelines. Detection of the PVL encoding genes and determination of the SCC*mec* type was done by polymerase chain reaction (PCR), while *spa* typing and cluster analysis was performed following DNA sequencing.

**Results:**

Of the 38 typeable MRSA isolates, 22 were of SCC*mec* type II and 16 were of SCC*mec* type IV. All SCC*mec* type II isolates were multidrug-resistant, lacked the PVL genes, and were of *spa* type t002 or closely related *spa* types. In contrast, the SCC*mec* type IV isolates were resistant to fewer classes of antimicrobial agents, often possessed the PVL genes (75.0%), and were of *spa* type t008 or closely related *spa* types.

**Conclusions:**

While the majority of ocular MRSA strains in this study fit the classical profile of HA-and CA-MRSA, some CA-MRSA isolates exhibited higher levels of antimicrobial resistance, which should be of particular concern to eye-care professionals. Furthermore, the apparent association of *spa* types and SCC*mec* types observed here warrants further investigation and suggests that *spa* typing may be useful in future HA- and CA-MRSA characterization studies.

## INTRODUCTION

*Staphylococcus aureus* is a leading human pathogen of significant clinical importance, responsible for a wide array of infections from superficial skin infections to more serious invasive infections, including pneumonia, septicemia, and endocarditis. It is also one of the most common ophthalmic pathogens recovered from conjunctivitis and other ocular infections.^1^ Since the isolation of the first methicillin-resistant *S. aureus* (MRSA) in 1961,^2^ the increasing prevalence of MRSA worldwide has become a growing concern,^3^,^4^ prompting the typing of *S. aureus* in order to support infection control measures, investigate suspected outbreaks, and evaluate nosocomial transmission.

Historically, MRSA pathogens were almost exclusively isolated from hospitals or hospital-associated facilities. However, there have been an increasing number of MRSA cases reported in individuals with no known risk factors for MRSA colonization, such as admission to a hospital, surgery, contact with a MRSA-colonized patient, intravenous drug use, or previous antibiotic exposure.^5^,^7^ These isolates, termed community-acquired MRSA (CA-MRSA), have become a global concern and have been found worldwide not only in the community setting but also in healthcare facilities.^8^ In fact, some hospitals have reported a predominance of CA-MRSA isolates over hospital-acquired MRSA (HA-MRSA) isolates.^9^,^10^ Although the term “acquired” implies that the location of transmission is known, the HA- and CA-designations have also been used to describe the phenotypic and molecular traits of MRSA isolates, as we have done in this study.

HA-MRSA strains, exemplified by the USA100 clone, are typically associated with nosocomial infections including bacteremia,^11^ whereas CA-MRSA strains, exemplified by the USA300 clone, have been more commonly associated with skin and soft tissue infections.^9^,^12^,^13^ The two groups are also distinguished by differences in their susceptibilities to antimicrobial agents, the composition of the gene cassette coding for methicillin resistance, and associated exotoxin profiles. Because CA-MRSA and HA-MRSA isolates are different with respect to virulence and antimicrobial susceptibility profiles, this information could be useful in the design of future strategies to prevent and treat ocular infections.

In contrast to HA-MRSA, which generally possess multiple antimicrobial resistance determinants and are thus multidrug-resistant, CA-MRSA are typically susceptible to non-β-lactam antibiotics.^14^ Resistance to β-lactam antibiotics, including methicillin, is conferred by a low affinity penicillin-binding protein (PBP) 2a, encoded by the *mecA* gene. The *mecA* gene is found on a mobile genetic element known as the “staphy-lococcal cassette chromosome *mec*” (SCCmec).^15^–^17^ To date, eight major variants of SCC*mec* (type I to VIII) have been identified,^18^ with SCC*mec* type II and type IV found predominantly in HA-MRSA and CA-MRSA, respectively.^19^–^21^ The Panton-Valentine leukocidin (PVL) genes, coding for a pore-forming cytotoxin known to cause tissue necrosis and leukocyte destruction, are frequently present in CA-MRSA and have been shown to be stable markers of CA-MRSA cases worldwide.^20^,^22^–^24^ In fact, CA-MRSA has been shown to be more virulent compared to HA-MRSA due to the presence of various virulence factors, such as PVL.^4^,^17^,^25^,^26^ Both SCC*mec* typing and detection of the PVL locus are useful tools for the molecular characterization of HA- and CA-MRSA isolates.

A different tool used for the typing of both MRSA and MSSA is single locus DNA sequencing of the *S. aureus* Protein A gene variable repeat region *(spa* typing). The *spa* gene contains a hypervariable region that differs in the number of repeats (1 to 23) and the number of base pairs (21 to 30) in each repeat (http://spaserver.ridom.de, accessed 20 Jul 2010).^27^,^28^ The nucle-otide composition of each distinct repeat is determined and subsequently given a *spa* type designation based on the unique succession of repeats. To date, pulsed-field gel electrophoresis (PFGE) is frequently used to determine clonal relationships between bacterial isolates. However, the method is cumbersome and PFGE results cannot be easily compared among multiple laboratories.^29^ In contrast, the *spa* typing method, along with the recently described *spa* grouping algorithm BURP (Based Upon Repeat Patterns), provides a rapid and accurate method to determine clonal relationships among *S. aureus* strains.^29^–^31^

While much attention has been paid to MRSA isolates from skin, soft tissue, and invasive infections, less is known about the prevalence and epidemiology of MRSA in infections of the eye. Since commensal bacteria from the skin and nasopharynx are often the source of ocular infections and CA-MRSA often cause skin and soft tissue infections, it was of interest to determine whether strains that have traits similar to those of HA-MRSA or those of CA-MRSA are more prevalent among drug-resistant isolates from ocular infections. As part of an ongoing study to characterize fluoroquinolone (FQ) resistance in ocular *S. aureus* isolates, we chose to further characterize such isolates with respect to the microbiological and molecular features of CA- and HA-MRSA isolated in the USA. Accordingly, SCC*mec* typing, the presence of the PVL gene, and antimicrobial susceptibility testing were used to characterize these strains for traits typical of either CA-MRSA or HA-MRSA. *Spa* typing was also performed to explore its use as a potential method for characterizing HA-MRSA and CA-MRSA strains.

## MATERIALS AND METHODS

### Bacterial Strains and Growth Conditions

As part of a separate study to characterize the molecular basis of high-level FQ resistance among staphylo-cocci, a total of 56 ocular *S. aureus* strains including MRSA and MSSA were obtained from Eurofins Medinet (Chantilly, Virginia, USA). Ocular isolates were collected between 2006 and 2008, representing 24 hospitals from 14 different U.S. states. Strains were isolated from one of four different ocular sources (aqueous fluid, vitreous fluid, conjunctiva, or cornea) from patients aged < 1 to 90 years (61% female). Care was taken to ensure that no duplicate isolates were included in this study.

All strains were grown for 18-24 hr at 37°C under ambient conditions. For genomic DNA extractions, bacteria were grown in Tryptic Soy Broth (Difco, Sparks, Maryland). Susceptibility testing was performed with Mueller-Hinton Broth II (Difco). ATCC 29213 (American Type Culture Collection [ATCC], Manassas, Virginia, USA) was the *S. aureus* quality control strain used for Clinical Laboratory and Standards Institute (CLSI) compliant susceptibility testing.^32^ Two MRSA strains, USA300 and USA600 (ATCC), were used as controls for SCC*mec* typing.

### Antimicrobial Susceptibility Testing

In vitro antimicrobial susceptibility testing for besifloxacin (BES), moxifloxacin (MXF), gatifloxa-cin (GAT), ciprofloxacin (CIP), levofloxacin (LVX), azithromycin (AZI), vancomycin (VAN), rifampin (RIF), clindamycin (CLI), tobramycin (TOB), erythromycin (ERY), tetracycline (TET), linezolid (LIN), and oxacillin (OXA) was performed according to CLSI guidelines.^32^ All agents were obtained in powder form from LKT Laboratories (St. Paul, Minnesota, USA), with the exception of BES, which was obtained from Bausch & Lomb Inc. (Rochester, New York, USA). In accordance with the manufacturer's recommendations, all agents were solubilized and diluted. The minimum inhibitory concentration (MIC) was reported as the lowest antimicrobial concentration that inhibited the visible growth of bacteria. Since susceptibility test interpretive criteria (breakpoints) have yet to be defined by the Food and Drug Administration (FDA) or CLSI for topical agents, systemic breakpoints were used, where available, to classify bacterial isolates as susceptible or resistant. Because besifloxacin was developed as an exclusively topical ophthalmic agent, no breakpoints are currently defined.

### *spa* Typing and BURP Cluster Analysis

Genomic DNA was extracted from pure bacterial cultures using the Wizard Genomic DNA Purification Kit (Promega, Madison, Wisconsin, USA) according to the manufacturer's instructions and was used as the template for polymerase chain reaction (PCR) amplification. The *spa* variable repeat region from each isolate was amplified using previously published oligonucleotide primers.^33^ PCR was carried out in a MyCycler thermal cycler (BioRad, Hercules, California, USA) in a 100 μl volume containing 0.4 μM primers (Integrated DNA Technologies, Coralville, Iowa, USA), 200 μM deoxynucleoside triphosphates (Omega Bio-Tek, Norcross, Georgia, USA), 1U of Vent DNA polymerase and its reaction buffer containing 2mM magnesium sulfate (New England Biolabs, Ipswich, Massachusetts, USA). The initial cycle of denaturation (15min at 94°C) preceded 30 cycles consisting of 0.5 min of denaturation at 94°C, 1 min of annealing at 59°C, and 1 min of elongation at 72°C, and was followed by a final cycle of elongation (10 min at 72°C). PCR products were purified using the EZNA Cycle Pure Kit (Omega Bio-Tek), and sequenced by ACGT Inc. (Wheeling, Illinois, USA). Clone Manager 9 (Sci-Ed Software, Cary, North Carolina, USA) was used for sequence analyses, and *spa* types were determined using the website (http://spaserver.ridom.de/) developed by Ridom GmbH and curated by SeqNet.org (http://SeqNet.org/).^28^ The BURP clustering tool in the Ridom StaphType 2.0.3 software package (Ridom GmbH, Würzburg, Germany) was used for *spa* type aligning and clustering.^31^

### Detection of Genes Encoding PVL

Genomic DNA was extracted as described above and used as the template for PCR amplification. A 945-bp region of the Panton-Valentine leukocidin genes *(lukF-PV* and *lukS-PV)* from each isolate was amplified using the oligonucleotide primers, *lukS* 5'-CCC ATT AGT ACA CAG TGG TTT CAA TC-3’ and *lukF* 5'-GTC CAG CAT TTA AGT TGC TTT GTC-3'. The primer sequences were designed from the published *S. aureus* strain USA300 (GenBank accession no. NC_007793) using Clone Manager 9 analysis software (Sci-Ed Software). PCR was carried out as described above in a 25 μl volume. The initial cycle of denaturation (10 min at 94°C) preceded 33 cycles consisting of 0.5 min of denaturation at 94°C, 0.5 min of annealing at 57°C, and 1 min of elongation at 72°C, and was followed by a final cycle of elongation (10min at 72°C).

### SCCmec Typing

Genomic DNA was extracted as described by Zhang et al.^34^ with modifications. Briefly, five to ten bacterial colonies were suspended in 50 μl of nuclease-free water (Promega) and heated at 99°C for 10 min. After cen-trifugation at 21,000 × *g* for 1 min, 2.5-9 μl of the supernatant was used as the template for PCR amplification. The SCC*mec* typing assay contained eight unique and specific pairs of primers as previously described^35^ for SCCmec types and subtypes I, II, III, IVa, IVb, IVc, IVd, and V. PCR was carried out as simplex reactions using conditions identical to those described above for PVL detection.

### Gel Electrophoresis

PCR amplicons were visualized using a UV light box after electrophoresis on 1% *(spa* typing) or 2% (detection of PVL genes and SCC*mec* typing) agarose gels containing 0.1 ug/ml ethidium bromide (Omega Bio-Tek).

## RESULTS

Published CLSI breakpoints for OXA^36^ were used to classify all ocular *S. aureus* isolates as either MSSA (n = 16) or MRSA (n = 40). With the exception of two MRSA isolates, where the SCC*mec* type could not be determined, the remaining 38 MRSA isolates were classified as either SCC*mec* type II (n=22) or type IV (n = 16) (Table 1). Two different subtypes of the SCC*mec* type IV cassette were found with subtype IVa identified in 15 (93.8%) of the type IV isolates and subtype IVb being found in a single isolate (6.3%). The PVL genes were absent in all SCC*mec* type II isolates, while 75.0% (12/16) of SCC*mec* type IV and 18.8% (3/16) of the MSSA isolates contained the PVL genes. Four different, but related, *spa* types were found among SCC*mec* type II isolates while five different, but related, *spa* types were found among SCC*mec* type IV isolates; MSSA was the most diverse with respect to *spa* type with 12 different types being observed.

**TABLE 1:** Summary of ocular *S. aureus* characteristics.

	MRSA		
	Type II	Type IV	MSSA
Number of isolates	22	16	16
Source of isolates by state (number of sites)	AL (2), FL (1), GA (1), ID (1), MA (2), MD (1), MI (3), NY (2), OH (2), PA (4), SC (1), TN (2)	FL (1), GA (1), ID (1), MA (1), MD (4), MI (1), NC (1), NY (2), OH (4)	AL (1), FL (2), GA (1), MA (2), MD (1), MI (1), MO (1), NY (3), OH (2), PA (2)
SCCmec type (n)	II (22)	IVa (15)	NA
		IVb(1)	
PVL positive, n (%)	0 (0.0%)	12 (75.0%)	3 (18.8%)
Number of *spa* types	4	5	12
% Resistant to 1+ drug class^a^	100.0%	100.0%	62.5%
% Resistant to 2+ drug classes	100.0%	93.8%	37.5%
% Resistant to 3+ drug classes	100.0%	62.5%	18.8%
% Resistant to 4+ drug classes	95.5%	6.3%	6.3%
% Resistant to 5+ drug classes	36.4%	0.0%	0.0%
% Resistant to 6+ drug classes	4.5%	0.0%	0.0%
% Resistant to 7+ drug classes	0.0%	0.0%	0.0%

a Drug classes tested included fluoroquinolones, macrolides, lincosamides, aminoglycosides, tetracyclines, rifamycins, glycopeptides, oxazolidinones, β-lactams.

Susceptibility testing was performed with 14 antimicrobial agents representing the following nine drug classes: fluoroquinolones (FQs), macrolides, lincos-amides, aminoglycosides, tetracyclines, rifamycins, glycopeptides, oxazolidinones, and β-lactams. All SCC*mec* type II isolates were resistant to at least three drug classes, with one isolate in this group exhibiting resistance to six different drug classes. Conversely, 62.5% of SCC*mec* type IV isolates were resistant to at least three drug classes, with no isolate in this group possessing resistance to more than four different drug classes.

The MIC_50_, MIC_90_, and MIC range values for all antimicrobial agents tested are presented in Table 2. One hundred percent of all *S. aureus* isolates were susceptible to the systemic (i.e., non-ophthalmic) agents RIF, VAN, and LIN. Additionally, 100% of the SCCmec type IV isolates were susceptible to CLI and TET while 87.5% and 93.8% of MSSA isolates and 54.5% and 95.5% of the SCCmec type II isolates were susceptible to these drugs, respectively. With the exception of BES, where the percentage of susceptible isolates cannot be determined due to the lack of any established resistance breakpoints for exclusively topical agents, all SCC*mec* type II isolates were resistant to the FQs (MXF, GAT, CIP, and LVX) and macrolides (AZI and ERY) tested. However, 37.5% and 6.3% of SCC*mec* type IV isolates were susceptible to all FQs and macrolides tested, respectively; 56.3% of MSSA isolates were susceptible to the macrolides and early generation FQs (CIP and LVX), while 62.5% of MSSA isolates were susceptible to the subsequent generation of FQ agents (GAT and MXF). While only 13.6% of SCC*mec* type II isolates were susceptible to TOB, 93.8% of SCC*mec* type IV isolates and 81.3% of MSSA isolates exhibited susceptibility to this aminoglycoside.

**TABLE 2:** Antimicrobial susceptibilities of ocular *S. aureus,* including 16 MSSA, 22 MRSA *SCCmec* type II, and 16 MRSA *SCCmec* type IV isolates.

		MIC (ng/ml)			
Agent	Organism	Range	50%	90%	% Susceptible
Besifloxacin	MSSA	0.016-4	0.03	4	NA^a^
	MRSA (type II)	0.5-8	4	4	NA^a^
	MRSA (type IV)	0.016-1	0.25	0.5	NA^a^
Moxifloxacin	MSSA	0.03-64	0.06	32	62.5
	MRSA (type II)	2-64	32	64	0
	MRSA (type IV)	0.016-2	2	2	37.5
Gatifloxacin	MSSA	0.06-64	0.125	32	62.5
	MRSA (type II)	2-128	64	128	0
	MRSA (type IV)	0.031-2	2	2	37.5
Ciprofloxacin	MSSA	0.125-256	0.5	256	56.3
	MRSA (type II)	32-256	256	256	0
	MRSA (type IV)	0.125-64	16	32	37.5
Levofloxacin	MSSA	0.125-512	0.25	512	56.3
	MRSA (type II)	8-1024	512	512	0
	MRSA (type IV)	0.125-8	4	8	37.5
Azithromycin	MSSA	0.5-> 256	0.5	>256	56.3
	MRSA (type II)	>256	>256	>256	0
	MRSA (type IV)	0.5-> 256	128	128	6.3
Erythromycin	MSSA	0.25-> 256	0.5	>256	56.3
	MRSA (type II)	>256	>256	>256	0
	MRSA (type IV)	0.25-64	64	64	6.3
Clindamycin	MSSA	0.063-> 256	0.125	>256	87.5
	MRSA (type II)	0.063-> 256	0.25	>256	54.5
	MRSA (type IV)	0.063-0.125	0.063	0.063	100
Tobramycin	MSSA	0.25-256	0.5	256	81.3
	MRSA (type II)	0.5-256	256	256	13.6
	MRSA (type IV)	0.5-32	1	2	93.8
Tetracycline	MSSA	0.5-32	0.5	4	93.8
	MRSA (type II)	0.25-64	0.5	0.5	95.5
	MRSA (type IV)	0.25-0.5	0.5	0.5	100
Rif ampin	MSSA	0.002-0.016	0.008	0.008	100
	MRSA (type II)	0.004-0.125	0.008	0.008	100
	MRSA (type IV)	0.004-0.008	0.004	0.008	100
Vancomycin	MSSA	1	1	1	100
	MRSA (type II)	1	1	1	100
	MRSA (type IV)	0.5-1	1	1	100
Linezolid	MSSA	2-4	2	4	100
	MRSA (type II)	2-4	4	4	100
	MRSA (type IV)	2	2	2	100
Oxacillin	MSSA	0.25-0.5	0.25	0.5	100
	MRSA (type II)	>8	>8	>8	0
	MRSA (type IV)	4->8	>8	>8	0

a Since besifloxacin was developed as an exclusively topical ophthalmic agent, no breakpoints exist.

Rifampin was the most potent antimicrobial agent tested for all *S. aureus* isolates with MIC_90_ values of 0.008 ug/ml. Among the FQ agents tested, BES was the most potent agent, with MIC_90_ values of 4 ng/ml for SCC*mec* type II and MSSA isolates and 0.5μg/ml for SCC*mec* type IV isolates. For all FQ agents tested, the MSSA isolates displayed the broadest MIC ranges. The highest MIC_90_ values observed among the SCC*mec* type II isolates and MSSA isolates were 512μg/ml for LVX, > 256μg/ml for AZI, ERY, and CLI, and 256μg/ml for TOB and CIP. In contrast the highest MIC_90_ values for SCCmec type IV isolates were 128μg/ml for AZI, 64μg/ml for ERY, and 32μg/ml for CIP.

The *spa* typing data presented in Table 3 revealed that all 22 SCC*mec* type II isolates, representing four different *spa* types, occurred within the same BURP cluster (spa-CC002) with *spa* type t002 observed most frequently (81.8%; 18/22). Similarly, the 16 SCC*mec* type IV isolates, representing five different *spa* types, occurred within the same BURP cluster (spa-CC008) with *spa* type t008 observed most frequently (75.0%; 12/16). In contrast, the 16 MSSA isolates displayed by far the most diversity in terms of the number of different *spa* types found; with the exception of *spa* type t002, which occurred five times, no other type was represented more than once. The 12 different *spa* types found among the MSSA isolates comprised four BURP clusters (spa-CC002, spa-CC008, spa-CC084, and spa-CC240/773) in addition to three singletons.

**TABLE 3:** *spa* clusters, *spa* types, repeat patterns, and (n) number for ocular *S. aureus.*

MRSA SCC*mec* type II (n=22)		
*spa CC002 cluster*		
t002	26-23-17–34-17–20-17-12–17-16	(18)
t045	26-17–20-17-12-17-16	(1)
t067	26-23-17–34-17–20-17-12-17	(2)
t242	26-23-17-13-17-20-17-12-17-16	(1)

MRSA SCC*mec* type IV (n=16)		

*spa CC008 cluster*		
t008	11–19-12–21-17–34-24–34-22–25	(12)
t024	11–12–21-17–34-24–34-22–25	(1)
t334	11–12–21-17–34-22–25	(1)
t622	11–19-12–21-17–34-22–25	(1)
t1578	11–19-12-21-17–34-24–34-17	(1)

MSSA (n=16)		

*spa CC002 cluster*		
t002	26-23-17–34-17–20-17-12-17-16	(5)
t242	26-23-17-13–17–20-17-12-17-16	(1)
*spa CC008 cluster*		
t008	11–19-12-21-17-34-24–34-22-25	(1)
t064	11–19-12-05-17-34-24-34-22-25	(1)
*spa CC084 cluster*		
t084	07-23-12–34–34-12–12–23-02-12–23	(1)
t346	07-23-12-34-12-12–23-02-12–23	(1)
t491	26-23-12-34-34-12-12–23-02-12-23	(1)
*spa CC240/773 cluster*		
t240	04-44-33-31-12-16–34-16-12-22–34	(1)
t773	04-44-33-31-16-12–25-22–34	(1)
*Singletons*		
t004	09-02-16-13-13-17-34-16-34	(1)
t005	26-23-13–23-31-05-17–25-17–25-16–28	(1)
t160	07-23-21-24–33-22-17	(1)

## DISCUSSION

Molecular and microbiological characterizations were conducted to determine whether ocular isolates have traits characteristic for CA-MRSA or HA-MRSA. To our knowledge, no SCC*mec* typing study^13^ has been conducted on an exclusively ocular set of MRSA isolates. Because these isolates were also part of separate FQ resistance characterization studies among ocular isolates, a substantial fraction of strains tested here (41/56) had elevated FQ MIC values and therefore may not be fully representative of ocular MRSA in general. Nevertheless, because most ocular infections are treated empirically this report of multidrug-resistant strains isolated from the eye with genetic traits of both CA-MRSA and HA-MRSA should be of interest to the ophthalmic community.

The results of the current study showed that all 38 typeable ocular MRSA isolates tested could be classified as either SCC*mec* type II or SCC*mec* type IV, the predominant cassette types of HA-MRSA and CA-MRSA, respectively.^21^,^37^ Consistent with previous reports,^38^ all the ocular SCC*mec* type II strains examined here exhibited traits typical for HA-MRSA, including the absence of the PVL toxin and resistance to multiple drug classes (Table 1). Over 95% of the SCC*mec* type II isolates were resistant to at least four different classes of antimicrobial agents and MIC_50_ and MIC_90_ values against such agents were elevated in comparison to values observed among the SCC*mec* type IV isolates. The increased resistance to non β-lactam antibacterials found in the SCC*mec* type II isolates may be partly due to the fact that these cassettes contain a variety of additional drug resistance gene elements not found within SCC*mec* type IV cassettes.^39^

CA-MRSA isolates have usually been defined as containing SCC*mec* type IV cassettes, expressing PVL, and exhibiting susceptibility to non-β-lactam antimicrobial agents.^21^,^40^ In this study, 16 MRSA isolates were characterized as SCC*mec* type IV isolates and of these 75% were found to contain the PVL genes. In contrast to the SCC*mec* type II isolates, which all showed high level resistance to several classes of antimicrobial agents, all SCC*mec* type IV isolates shared only resistance to β-lactam agents. There were, however, high levels of resistance to other drug classes observed; among the SCC*mec* type IV isolates, susceptibility to the FQ and macrolide classes was only 37.5% and 6.3%, respectively.

Recently, in a large MRSA surveillance study conducted in San Francisco, Diep et al. found that almost 90% of their USA300 strains (n= 188) were resistant to ERY, over 60% were resistant to CIP, over 24% were resistant to TET, and over 10% were resistant to CLI.^41^ These data, taken together with our resistance data, indicate that resistance to non-β-lactam antibiotics might be increasing among SCC*mec* type IV isolates. The increased resistance and the fact that 25% of our SCC*mec* type IV isolates did not produce PVL, suggest that the criteria for classifying a MRSA isolate as either CA- or HA-MRSA are blurring. Nonetheless, the MIC_90_ values for all antimicrobial agents were equal to or lower for SCC*mec* type IV isolates than for SCC*mec* type II isolates.

PFGE or *spa* typing can reveal clonal relationships among MRSA isolates. The most observed types in this study were *spa* types t008 and t002 representing the clones USA300 and USA100, respectively. These *spa* types have been frequently reported as common *spa* types found in large surveillance studies worldwide.^42^–^44^ In the relative global frequencies database on the Ridom SpaServer website, *spa* types t003 (12.5%), t032 (10.7%), t008 (6.6%), and t002 (5.9%) are listed as the most commonly isolated *spa* types (http://spaserver.ridom.de/frequencies.shtml, accessed 07 Jun 2010). Although the Ridom database does contain isolates from all over the world, the data set is dominated by European isolates. For example, our analysis of the Ridom database revealed that of the 50 most frequent *spa* types (those with a prevalence of 0.25% or higher), 95.5% of the 59,811 isolates with country of origin listed were from Europe, while only 1.5% originated in the United States. The most common *spa* types among the 898 U.S. isolates were t008 (69.7%), t002 (11.8%), t064 (4.7%), t045 (2.8%), t024 (2.0%), and t242 (1.6%); the same six *spa* types were also identified among the 54 ocular isolates described here. These data support the hypothesis that the *spa* types of ocular *S. aureus* are similar to those isolated from other body sites.

All MRSA isolates containing the SCC*mec* type II element were either of *spa* type t002 or one of three similar *spa* types that belong to cluster spa-CC002. Type t002 was the founder of this cluster and was, with 18 isolates, the most prevalent *spa* type. Similarly, all strains containing a SCC*mec* type IV element were either *spa* type t008 or one of four *spa* types that are part of the spa-CC008 cluster. Type t008 was the founder of the spa-CC008 cluster and was, with 12 isolates, the second most prevalent *spa* type among MRSA isolates.

*Spa* types t002 and t242 (cluster spa-CC002) and t008 (cluster spa-CC008) were present in the MRSA and the MSSA groups. Several previous reports have documented the in vivo conversions of clinical MSSA to MRSA and vice versa.^45^,^46^ The acquisition or loss of DNA encoding the SCC*mec* cassette may occur more frequently in SCC*mec* type IV isolates than in SCC*mec* type II isolates, presumably due to the smaller, more mobile SCC*mec* type IV cassette.^19^,^21^,^47^ It remains to be determined if an ancestral strain acquired a specific SCC*mec* element and then diversified into clones with similar *spa* types, or if related strains independently converted from MSSA to MRSA by integrating SCC*mec* elements.^48^

In conclusion, the molecular characterization and, to some extent, the antimicrobial phenotypes of the MRSA isolates tested in this study demonstrate that SCC*mec* type II and SCC*mec* type IV isolates generally fit the classical definition of HA- and CA-MRSA strains, respectively. In contrast to MRSA, MSSA isolates were more diverse in their PVL, *spa* type, and antimicrobial susceptibility profiles, whereas the MRSA isolates were less varied with respect to these traits. In particular, the presence of a dominant *spa* type and the similarity between *spa* repeats among isolates with the same SCC*mec* type suggests that *spa* typing may be an additional useful tool when molecularly investigating and classifying ocular MRSA isolates as either CA- or HA-MRSA.
